# Eviction Moratoria Expiration and COVID-19 Infection Risk Across Strata of Health and Socioeconomic Status in the United States

**DOI:** 10.1001/jamanetworkopen.2021.29041

**Published:** 2021-08-30

**Authors:** Sebastian Sandoval-Olascoaga, Atheendar S. Venkataramani, Mariana C. Arcaya

**Affiliations:** 1 Department of Urban Studies and Planning, Massachusetts Institute of Technology, Cambridge; 2 OptumLabs Visiting Fellow, Eden Prairie, Minnesota; 3 Perelman School of Medicine, University of Pennsylvania, Philadelphia; 4 Leonard Davis Institute of Health Economics, University of Pennsylvania, Philadelphia

## Abstract

**Importance:**

Housing insecurity induced by evictions may increase the risk of contracting COVID-19.

**Objective:**

To estimate the association of lifting state-level eviction moratoria, which increased housing insecurity during the COVID-19 pandemic, with the risk of being diagnosed with COVID-19.

**Design, Setting, and Participants:**

This retrospective cohort study included individuals with commercial insurance or Medicare Advantage who lived in a state that issued an eviction moratorium and were diagnosed with COVID-19 as well as a control group comprising an equal number of randomly selected individuals in these states who were not diagnosed with COVID-19. Data were collected from OptumLabs Data Warehouse, a database of deidentified administrative claims. The study used a difference-in-differences analysis among states that implemented an eviction moratorium between March 13, 2020, and September 4, 2020.

**Exposures:**

Time since state-level eviction moratoria were lifted.

**Main Outcomes and Measures:**

The primary outcome measure was a binary variable indicating whether an individual was diagnosed with COVID-19 for the first time in a given week with *International Statistical Classification of Diseases and Related Health Problems, Tenth Revision* code U07.1. The study analyzed changes in COVID-19 diagnosis before vs after a state lifted its moratorium compared with changes in states that did not lift it. For sensitivity analyses, models were reestimated on a 2% random sample of all individuals in the claims database during this period in these states.

**Results:**

The cohort consisted of 509 694 individuals (254 847 [50.0%] diagnosed with COVID-19; mean [SD] age, 47.0 [23.6] years; 239 056 [53.3%] men). During the study period, 43 states and the District of Columbia implemented an eviction moratorium and 7 did not. Among the states that implemented a moratorium, 26 (59.1%) lifted their moratorium before the US Centers for Disease Control and Prevention issued their national moratorium, while 18 (40.1%) maintained theirs. In a Cox difference-in-differences regression model, individuals living in a state that lifted its eviction moratorium experienced higher hazards of a COVID-19 diagnosis beginning 5 weeks after the moratorium was lifted (hazard ratio [HR], 1.39; 95% CI, 1.11-1.76; *P* = .004), reaching an HR of 1.83 (95% CI, 1.36-2.46; *P* < .001) 12 weeks after. Hazards increased in magnitude among individuals with preexisting comorbidities and those living in nonaffluent and rent-burdened areas. Individuals with a Charlson Comorbidity Index score of 3 or greater had an HR of 2.37 (95% CI, 1.67-3.36; *P* < .001) at the end of the study period. Those living in nonaffluent areas had an HR of 2.14 (95% CI, 1.51-3.05; *P* < .001), while those living in areas with a high rent burden had an HR of 2.31 (95% CI, 1.64-3.26; *P* < .001).

**Conclusions and Relevance:**

The findings of this difference-in-differences analysis suggest that eviction-led housing insecurity may have exacerbated the COVID-19 pandemic.

## Key Points

**Question:**

Is lifting a state-level eviction moratorium associated with the risk of individuals in that state being diagnosed with COVID-19?

**Findings:**

In this cohort study of 509 694 individuals living in the United States, a difference-in-differences survival analysis found that residents in states that lifted eviction moratoria had an increased risk of receiving a COVID-19 diagnosis 12 weeks after the moratorium was lifted relative to residents in states where moratoria remained in place. These associations increased over time, particularly among individuals with more comorbidities and lower socioeconomic status.

**Meaning:**

These findings suggest that eviction-led housing insecurity may have exacerbated the COVID-19 pandemic.

## Teaser

This cohort study estimates the association of lifting US state–level eviction moratoria with the risk of being diagnosed with COVID-19.

## Introduction

On September 4, 2020, the US Centers for Disease Control and Prevention (CDC) enacted a national eviction moratorium because “the evictions of tenants could be detrimental to public health control measures to slow the spread of the virus that causes COVID-19.”^1^ The moratorium came at a time when an estimated 47.0% of individuals in renter-occupied housing behind on their payments were likely to leave their homes due to eviction,^2^ sequalae of the United States’ long-standing housing-affordability crisis and the COVID-19 pandemic’s impact on employment and income.^3^

A growing body of evidence suggests that eviction activity may be associated with increased COVID-19 infection rates. For example, a study^4^ using ecologic data on COVID-19 infection rates and timing of state-level eviction bans found that COVID-19 rates increased after eviction moratoria expired. Other investigations using simulations have since found that households experienced an increased risk of infection not just due to personal experiences but also due to spillover from the transmission processes amplified by community evictions.^5^

However, limitations in public health surveillance data do not allow for exploration of differential policy effects based on individual-level health and socioeconomic characteristics. Understanding whether expiring eviction moratoria are particularly dangerous for people and local geographies that have already experienced disproportionate effects of the pandemic, including individuals with preexisting health problems and low-income communities, could help to inform how nonpharmaceutical interventions are deployed with an equity focus. For example, shelter-in-place orders, which protect professional class workers but not essential workers from occupational exposures, likely have different distributional impacts than do eviction moratoria, which we expect to disproportionately protect lower-income and rent-burdened populations and places.

We used detailed health care claims data from a large national database in the United States to conduct what we believe to be the first individual-level analysis of how eviction policy affects the hazard of a COVID-19 diagnosis within health and neighborhood-level socioeconomic strata. We used a difference-in-differences research strategy to compare changes in the risk of being diagnosed with COVID-19 before and after the lifting of state-level eviction moratoria vs the same changes in states that maintained these moratoria. We also assessed how associations between eviction moratoria and the risk of COVID-19 diagnosis varied by an individual’s Charlson Comorbidity Index (CCI) score as well as by zip code–level poverty and rent burden prevalence, to test the hypotheses that (1) individuals with poorer baseline health, as measured by the CCI, will experience higher risk of infection after moratoria are allowed to expire because baseline health status and eviction risk are both socially patterned and (2) individuals in low-income and rent-burdened communities will be at heightened risk of infection after expiring moratoria due to higher risk of exposure to eviction-related COVID-19 transmission driven by local evictions and subsequent crowding.

## Methods

### Data and Study Population

We used deidentified administrative claims data from the OptumLabs Data Warehouse (OLDW), which includes medical claims and enrollment records for individuals with commercial insurance and Medicare Advantage (MA) but does not include those with Medicare fee-for-service or Medicaid. The database contains health information on nearly 200 million enrollees, representing a mixture of ages, ethnicities, and geographical regions across the United States.^6^ The Massachusetts Institute of Technology Committee on the Use of Humans as Experimental Subjects exempted this study from review and the requirement for informed consent because it involved private deidentified information. This study adheres to the Strengthening the Reporting of Observational Studies in Epidemiology (STROBE) reporting guideline for cohort studies.

Our study cohort, organized as an individual-weekly panel, included all individuals with commercial insurance and MA who (1) lived in a state in which an eviction moratorium was issued^7^ and (2) were diagnosed with COVID-19 during the period between the week the state first issued its eviction moratorium and the week the CDC issued the nationwide eviction moratorium (n = 254 847). Our primary analytic sample (ie, balanced sample) also included a control group comprising an equal number of randomly selected individuals who were not diagnosed with COVID-19 in the same time period and states. We focused on an analytic sample that contained all individuals with a COVID-19 diagnosis to increase the statistical power to detect differences in the association of the eviction moratorium policy with COVID-19 diagnosis by stratifying variables.

### Outcome, Exposure, and Covariates

Our primary outcome measure was a binary variable that varied by week, indicating whether the individual was diagnosed for the first time in that week with *International Statistical Classification of Diseases and Related Health Problems, Tenth Revision *(*ICD-10*) code U07.1. Our exposure variable enabled an event-time study, with time centered at the week a state’s court, governor, or legislature lifted its eviction moratorium for the first time.^7^

We included the following covariates: number of weeks that had passed since the issuance of a state mask mandate, a stay-at-home or shelter-in-place order, the closure of schools, the state began lifting business restrictions, and the reopening of movie theaters^7^; weekly county-level COVID-19 cases lagged by 2 weeks^8^; weekly state-level COVID-19 tests lagged by 2 weeks^8^; zip code–level poverty rate^9^; week and state fixed effects; an individual’s sex, age (centered at 65 years), type of insurance (commercial or MA), and latest industry of employment; and whether the individual had a Z code, ie, a diagnosis of problems related to unemployment (*ICD-10* code, Z56), problems related to housing and economic circumstances (*ICD-10* code Z59), or problems related to bereavement (*ICD-10* code, Z64.4) before 2020. We included an individual’s CCI score as a baseline measure of global comorbidity before the pandemic and the study period began.^10^ We used an individual’s available claims history from 2017 to 2020 to obtain a continuous positive index that we stratified into 4 categories (0, 1, 2, or ≥3).

### Statistical Analysis

To study the association between lifting the eviction moratorium on the hazard of being diagnosed with COVID-19 in a given week, we used a Cox regression model with time-dependent covariates in an event-time type specification.^11,12^ This approach models the weekly probability of being diagnosed with COVID-19 at a given period conditional on having been observed without a positive diagnosis previously, where the treatment is defined as lifting the eviction moratorium and treated individuals are compared with individuals living in states that had not yet lifted their moratoria (eMethods 1 in the Supplement).

This study used the time from when individuals entered the study until either a COVID-19 diagnosis or the end of the study period, just like in a classic Cox analysis. Unlike a standard Cox model, however, we also made use of information on time since the treatment occurred (ie, since the eviction moratorium was lifted) for the individuals considered treated. This method allows us to understand whether the association between expiring eviction moratoria and a COVID-19 diagnosis changed over time, which is useful when studying events that develop exponentially, such as epidemics, while also relaxing the proportional hazards assumption.

The causal identifying assumption is that COVID-19 diagnosis risk in exposed states would have continued along the same trajectories in the absence of exposure.^11^ We cannot directly test this assumption. Nevertheless, potential violations can be probed by examining outcome trends for event weeks before lifting the eviction moratorium. We formally tested this through a joint significance χ^2^ test simultaneously of all the terms before the eviction moratorium was lifted.

The primary analysis focused on being diagnosed with COVID-19 in the entire sample. We also conducted analyses stratifying by a series of individual- and zip code–level risk factors that could plausibly modify the association of expiring eviction moratoria with COVID-19 risk as time since treatment passed. Specifically, we stratified our sample by an individual’s CCI score; by zip code–level poverty rate, measured by whether the percentage of individuals living below the poverty line was greater or less than 10%, a cut point commonly used to designate low-poverty neighborhoods^13,14^; and by zip code–level rent-burden prevalence, measured by whether more or less than half of households renting a unit were spending at least 30% of their household income on rent, a cut point that divided our sample roughly in half and allowed us to compare higher and lower rent-burdened places with equal sample size. We tested whether the association in these subgroups increased as time since treatment passed through a joint significance χ^2^ test. For all models, we plotted fully adjusted hazard ratios (HRs) with 95% CIs by week, adjusted for clustering at the state and week level, centered on the week the eviction moratorium expired. Each adjusted HR shows the difference in outcomes for leads and lags of lifting the eviction moratoria relative to a reference week (ie, the week a state lifted their moratorium) and relative to all states that did not lift their eviction moratorium during the reference period.

To provide cumulative differences in the hazard of COVID-19 infection, we calculated survival curves derived from our models (eMethods 2 in the Supplement). We computed 2 opposing counterfactual scenarios: (1) every state that implemented an eviction moratorium maintained it throughout the study period and (2) every state that implemented an eviction moratorium lifted it on week 17. We chose week 17 because it was the first week a state lifted its eviction moratorium (Table 1).

**Table 1.** US States by Eviction Moratorium Implementation and Lifting Status

State	Eviction moratorium		Week of the year the moratorium was first lifted
	Implemented	Lifted	
Alabama	Yes	Yes	23
Alaska	Yes	Yes	27
Arizona	Yes	No	NA
Arkansas	No	NA	NA
California	Yes	No	NA
Colorado	Yes	Yes	25
Connecticut	Yes	No	NA
Delaware	Yes	Yes	27
District of Columbia	Yes	No	NA
Florida	Yes	No	NA
Georgia	No	NA	NA
Hawaii	Yes	No	NA
Idaho	Yes	Yes	19
Illinois	Yes	No	NA
Indiana	Yes	Yes	34
Iowa	Yes	Yes	23
Kansas	Yes	Yes	23
Kentucky	Yes	Yes	35
Louisiana	Yes	Yes	25
Maine	Yes	Yes	32
Maryland	Yes	Yes	31
Massachusetts	Yes	No	NA
Michigan	Yes	Yes	30
Minnesota	Yes	No	NA
Mississippi	Yes	Yes	23
Missouri	No	NA	NA
Montana	Yes	No	NA
Nebraska	Yes	Yes	23
Nevada	Yes	No	NA
New Hampshire	Yes	Yes	27
New Jersey	Yes	No	NA
New Mexico	Yes	No	NA
New York	Yes	No	NA
North Carolina	Yes	Yes	26
North Dakota	Yes	Yes	17
Ohio	No	NA	NA
Oklahoma	No	NA	NA
Oregon	Yes	No	NA
Pennsylvania	Yes	No	NA
Rhode Island	Yes	Yes	27
South Carolina	Yes	Yes	21
South Dakota	No	NA	NA
Tennessee	Yes	Yes	23
Texas	Yes	Yes	21
Utah	Yes	Yes	21
Vermont	Yes	No	NA
Virginia	Yes	Yes	21
Washington	Yes	No	NA
West Virginia	Yes	Yes	21
Wisconsin	Yes	Yes	22
Wyoming	No	NA	NA

Abbreviation: NA, not applicable.

In sensitivity analyses, we estimated every model with a random sample of all the individuals in the OLDW to ensure that our primary design did not introduce selection bias by choosing individuals by our outcome.^15^ Given the size of our original database and our computational limit, we worked with a 2% random sample. We overlaid these results on those calculated from the same model but with the balanced sample to assess for evidence of bias from our sample selection design. We also conducted the same exercise stratifying for the individual- and zip code–level risk factors previously described. Finally, we assessed whether expiring eviction moratoria were associated with an increase in an individual’s probability of eviction by estimating our main models’ expiring moratoria on the hazard of a zip code change in our claims data, a crude proxy for mobility.

All analyses were conducted in Stata version 16.1 (StataCorp). Statistical significance was set at *P* < .05, and all tests were 2-tailed. Claims data were extracted and processed using DbVisualizer version 10.0.15.

## Results

### Study Sample

Our study sample resided in 43 states and the District of Columbia because 7 states did not implement an eviction moratorium during our study period. (Table 1). These states accounted for 88.8% of the total US population in 2019^9^ and 89.6% of the US COVID-19 cases during the study period.^8^ Overall, 18 states (40.9%) never lifted their eviction moratorium during the study period so were included in the control group. The remaining 26 states (59.1%) functioned as the treatment group.

During the study period, our sample included 9 475 897 individual-week observations for 509 694 individuals (254 847 [50.0%] diagnosed with COVID-19; mean [SD] age, 47.0 [23.6] years; 239 056 [53.3%] men). Baseline demographic, health, and socioeconomic characteristics were similar in exposed vs unexposed states (Table 2), although there were higher COVID-19 diagnoses in states that lifted their eviction moratoria. Individuals were exposed to lifting the eviction moratorium from week 17 to 35 (eFigure 1 in the Supplement).

**Table 2.** Baseline Characteristics of Individuals Included in the Estimation Sample, Stratified by Exposure Status

Baseline characteristics	Individuals by whether the state lifted the eviction moratorium, No. (%)	
	No (265 359 individuals; 18 states)	Yes (244 335 individuals; 26 states)
COVID-19 diagnosis	141 050 (53.15)	113 797 (46.57)
Age, mean (SD), y	47.88 (22.94)	45.02 (22.37)
Sex		
Male	123 961 (46.72)	115 095 (47.12)
Female	141 359 (53.28)	129 123 (52.88)
Insurance		
Commercial	190 935 (71.95)	183 716 (75.19)
Medicare Advantage	74 424 (28.05)	60 619 (24.81)
Charlson Comorbidity Index score, mean (SD)	0.69 (1.10)	0.58 (1.02)
Flag		
Unemployment	288 (0.10)	247 (0.10)
Housing and economic circumstances	326 (0.12)	352 (0.14)
Bereavement	383 (0.14)	426 (0.17)
Zip code, mean (SD), %		
Poverty rate^a^	11.31 (7.39)	12.51 (8.07)
Rent burden prevalence^b^	50.27 (10.30)	45.79 (9.85)

^a^ Percentage of individuals living below the poverty line at the zip code level where the individual lives.

^b^ Percentage of households renting a unit and spending at least 30% of their household income in rent where the individual lives.

### Eviction Moratoria Expiration and COVID-19 Risk

**Figure 1.**
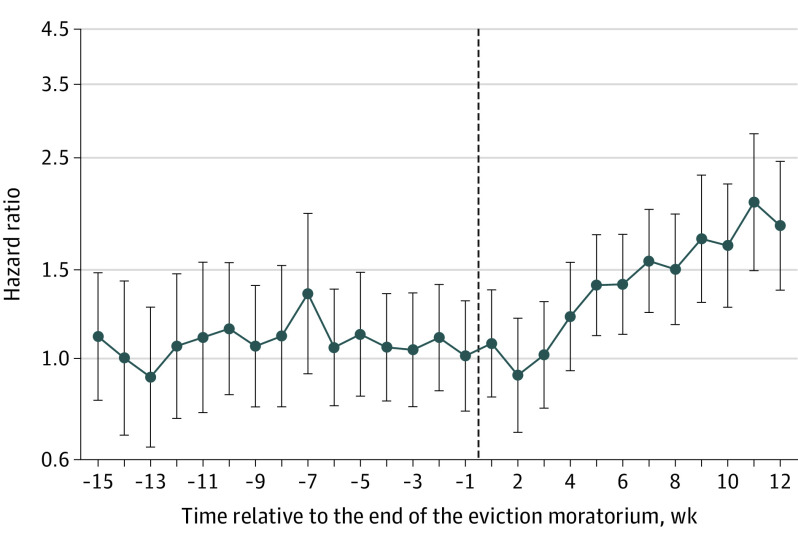
Event Study Estimates of the Association Between Lifting the Eviction Moratorium and Risk of COVID-19 Diagnosis Whiskers indicate 95% CIs.

Figure 1 plots the fully adjusted HRs of our main model. Before moratoria, there was no difference in trends in COVID-19 diagnosis risk between individuals in states lifting moratoria vs those keeping them in place, ie, we cannot reject the jointly null hypothesis in which every coefficient is equal to 1 before the moratoria (χ^2^_14_ = 5.35; *P* = .98), suggesting that in the absence of exposure, treatment and control groups would have continued along the same trajectory. Individuals living in states that lifted their eviction moratorium, relative to those living in states that never lifted their moratorium, were more likely to be diagnosed with COVID-19 beginning 5 weeks after the eviction moratorium was lifted (HR, 1.39; 95% CI, 1.11-1.76; *P* = .004) and reaching an HR of 1.83 (95% CI, 1.36-2.46; *P* < .001) at 12 weeks or longer. Looking at the cumulative difference in hazard of COVID-19 infection during the study period (eFigure 2 in the Supplement), we observed an average 2.4–percentage point (95% CI, 0.3-4.3 percentage points) higher probability of remaining in the study with no diagnosis of COVID-19 (*P* = .01) between the counterfactual scenarios in which every state lifted the eviction moratorium in week 17 of the year vs never lifting it.

**Figure 2.**
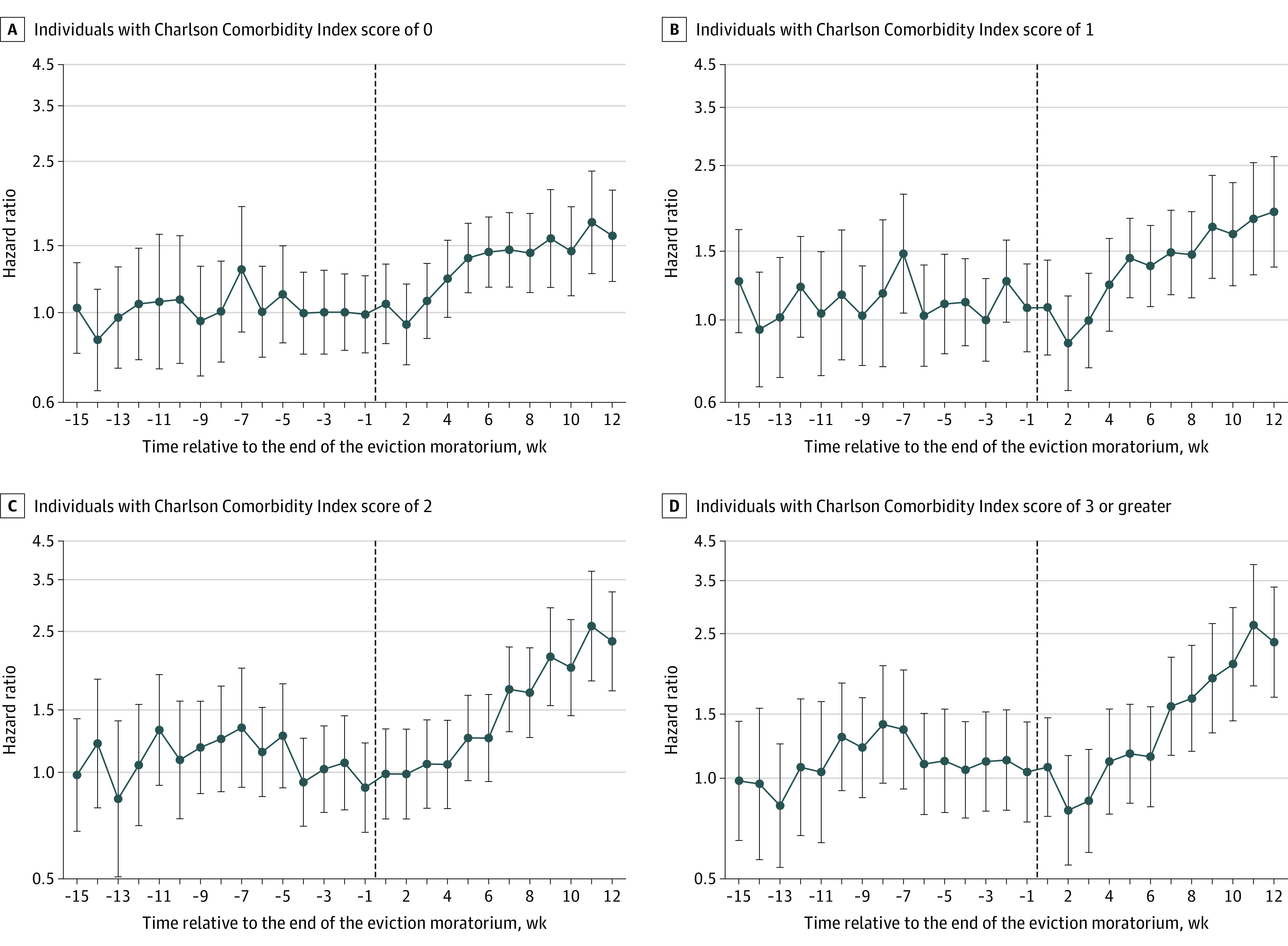
Event Study Estimates of the Association Between Lifting the Eviction Moratorium and Risk of COVID-19 Diagnosis, Stratified by Charlson Comorbidity Index Score Whiskers indicate 95% CIs.

Figure 2 plots the time-varying association between expiring eviction moratoria on individuals by baseline health strata, showing that associations increased with CCI score. The magnitude of the association increased as time since lifting an eviction moratorium passed for individuals with greater CCI scores. Individuals with a CCI of 3 or greater living in a state that lifted its eviction moratorium had an HR of 2.36 (95% CI, 1.67-3.36; *P* < .001) after 12 weeks compared with those living in a state that never lifted its moratorium. The healthiest group (ie, CCI score 0) was the only subgroup among the health strata where the associations plateaued after week 4 (χ^2^_7_ = 3.54; *P* = .83).

eFigure 3 in the Supplement plots the associations between moratoria and COVID-19 diagnosis risks by area-level poverty rates and rent burden, showing increasing associations for individuals in zip codes with higher levels of each. For areas with high poverty and a high rent burden, we can reject the null hypothesis that HRs after week 4 were equal for both groups (high poverty: χ^2^_7_ = 16.04; *P* = .02; high rent burden: χ^2^_7_ = 25.82; *P* <.001). Those living in nonaffluent areas had an HR of 2.14 (95% CI, 1.51-3.05; *P* < .001), while those living in areas with high rent burden had an HR of 2.31 (95% CI, 1.64-3.26; *P* < .001). However, we found statistically significant higher hazards for individuals living in low poverty and rent-burdened rate areas where the eviction moratoria were lifted compared with those living in control states, although they did not increase as time passed since lifting the eviction moratorium. In both the low-poverty and low rent-burdened rate models, we cannot reject the null hypothesis that HRs after week 4 were all equal (low-poverty: χ^2^_7_ = 5.79; *P* = 0.57; low rent burden: χ^2^_7_ = 4.35; *P* = .74).

### Sensitivity Analyses

Sensitivity analyses showed that the coefficients and confidence intervals of the balanced sample fell within the confidence intervals of the 2% random sample (eFigure 4 in the Supplement), providing evidence that our sample selection design did not bias our estimates. Furthermore, we found the same pattern when conducting the same analysis but using the CCI score and the poverty and rent-burden rates subgroups (eFigure 5 and eFigure 6 in the Supplement). We found no association between expiring eviction moratoria and whether an individual in our data set changed their zip code, suggesting that personal eviction experience was not the main mechanism by which expiring eviction moratoria caused increased COVID-19 hazard (eFigure 7 in the Supplement). Finally, while *ICD-10* Z codes are underused by practitioners,^16^ excluding these covariates did not affect results (eFigure 8 in the Supplement).

## Discussion

Using individual-level health care claims data, we found that lifting eviction moratoria was associated with an increase in the hazard of a COVID-19 diagnosis beginning 5 weeks after an eviction moratorium was lifted and persisting for at least 12 weeks after that point. As what we believe to be the first study on eviction policy and COVID-19 diagnoses to use individual-level data, we found that the hazards associated with lifting eviction bans increased with time among individuals with preexisting health problems. Our findings suggest that even individuals with no comorbidities were put at risk by expiring eviction moratoria after controlling for age and social factors, such as insurance type, occupational industry, history of unemployment, problems related to housing and economic circumstances, and area-level covariates. The result of the sensitivity analysis showing no association of expiring eviction moratoria on the hazard of individuals in this data set changing zip codes is consistent with previous findings in the literature, ie, an individual’s hazard of COVID-19 diagnosis was not just affected by personal experiences with eviction but also by spillovers from the transmission process created by evictions within a community.^5^ While previous ecological evidence showed that area-level COVID-19 incidence increases after eviction moratoria are lifted,^4^ these county-level analyses have not been able to answer the question of who, specifically, is put at risk by allowing evictions to occur during the COVID-19 pandemic.

Our findings are clear that the hazard of COVID-19 diagnosis increases for all individuals when eviction bans are allowed to expire, but that individuals with preexisting health problems and those living in areas with higher poverty or with a higher prevalence of rent-burdened households have disproportionately higher risk as time since ending the moratoria passes. As such, eviction moratoria should be thought of as a health equity intervention that has helped narrow the gap in risk between affluent and nonaffluent neighborhoods and between individuals based on preexisting health conditions, which, especially after age adjustments, are known to be associated with social determinants of health, including individual-level socioeconomic status and exposure to racism.^17^

Our investigation was designed as an event-time study^18,19^ that exploits the variation of some states implementing, lifting, or maintaining eviction moratoria while also including the timing of other COVID-19–related policy changes, such as mask mandates and school closures, that could have been timed in concert with eviction policy changes and could also affect COVID-19 hazard as well as with a set of individual- and area-level covariates to isolate the associations of expiring eviction moratoria. In the weeks before the eviction moratorium was lifted, there was no statistically significant difference in the HR of being diagnosed with COVID-19 between the states that lifted and did not lift their eviction moratoria, suggesting that the probability of being diagnosed with COVID-19 would have evolved similarly in all states absent the treatment. While we created a control balanced panel of individuals who were and were not diagnosed with COVID-19 during the observation period to provide power to the stratified analyses, we also conducted our main model on a 2% random sample of individuals who were not selected with regard to the outcome and found similar results, albeit with wider confidence intervals.

### Limitations

This study has limitations. First, we cannot rule out the chance that our associations could be explained by residual confounding, despite our methods and sensitivity analyses. Second, we relied on COVID-19 diagnoses as our outcomes. Thus, we are not including asymptomatic cases or individuals not interacting with the health sector despite having COVID-19. Third, our data set did not include information from individuals with Medicaid or those who are uninsured. However, since many of these individuals are at high risk of eviction^20^ and COVID-19, including them would strengthen the associations between expiring moratoria and COVID-19. Thus, our results should be considered a lower bound. Additionally, for privacy reasons, we did not have access to beneficiary race and ethnicity and so cannot describe the implications of allowing eviction moratoria to expire for racial and ethnic disparities in COVID-19 infection.

## Conclusions

In this cohort study with a difference-in-differences analysis, residents in states that lifted an eviction moratorium experienced increased risk of being diagnosed with COVID-19 compared with residents of states that maintained moratoria. The magnitude of associations increased over time after the moratoria were lifted among individuals with more comorbidities and for those living in higher poverty and rent-burdened zip codes. Beyond lessons for managing the COVID-19 pandemic as new variants spread, this study suggests that a housing policy that protects individuals with low income and/or more comorbidities can promote health equity and create protection for groups with more advantage.

## Notes

Supplement. **eFigure 1. **Trends in Share of Individuals Exposed to Treatment by the Week of the Year **eFigure 2. **Survival Curves on the Association Between Lifting the Eviction Moratorium and COVID-19 **eFigure 3. **Event Study Estimates of the Association Between Lifting the Eviction Moratorium and COVID-19, Stratified by Poverty and Rent-Burdenship Rate **eFigure 4. **Event Study Estimates of the Association Between Lifting the Eviction Moratorium and COVID-19, by Sample Design **eFigure 5. **Event Study Estimates of the Association Between Lifting the Eviction Moratorium and COVID-19, Stratified by Charlson Comorbidity Index and Sample Design **eFigure 6. **Event Study Estimates of the Association Between Lifting the Eviction Moratorium and COVID-19, Stratified by Poverty and Rent-Burdenship Rate and Sample Design **eFigure 7. **Event Study Estimates of the Association Between Lifting the Eviction Moratorium and Changing Zip Code Address **eFigure 8. **Event Study Estimates of the Association Between Lifting the Eviction Moratorium and COVID-19, With and Without Z Codes **eMethods 1. **Event-Time Study Model Specification **eMethods 2.** Survival Curves
eReferences.
