# Supplementary material for: Eviction Moratoria Expiration and COVID-19 Infection Risk Across Strata of Health and Socioeconomic Status in the United States
Source: JAMA Netw Open. 2021 Aug 30;4(8):e2129041. doi: 10.1001/jamanetworkopen.2021.29041 (PMC8406080; doi:10.1001/jamanetworkopen.2021.29041)
Supplement: Supplement. — eFigure 1. Trends in Share of Individuals Exposed to Treatment by the Week of the Year eFigure 2. Survival Curves on the Association Between Lifting the Eviction Moratorium and COVID-19 eFigure 3. Event Study Estimates of the Association Between Lifting the Eviction Moratorium and COVID-19, Stratified by Poverty and Rent-Burdenship Rate eFigure 4. Event Study Estimates of the Association Between Lifting the Eviction Moratorium and COVID-19, by Sample Design eFigure 5. Event Study Estimates of the Association Between Lifting the Eviction Moratorium and COVID-19, Stratified by Charlson Comorbidity Index and Sample Design eFigure 6. Event Study Estimates of the Association Between Lifting the Eviction Moratorium and COVID-19, Stratified by Poverty and Rent-Burdenship Rate and Sample Design eFigure 7. Event Study Estimates of the Association Between Lifting the Eviction Moratorium and Changing Zip Code Address eFigure 8. Event Study Estimates of the Association Between Lifting the Eviction Moratorium and COVID-19, With and Without Z Codes eMethods 1. Event-Time Study Model Specification eMethods 2. Survival Curves eReferences. [file jamanetwopen-e2129041-s001.pdf]

Supplemental Online Content

Sandoval-Olascoaga S, Venkataramani AS, Arcaya MC. Eviction moratoria expiration and COVID-19 infection risk across strata of health and socioeconomic status in the United States. *JAMA Netw Open.* 2021;4(8):e2129041. doi:10.1001/jamanetworkopen.2021.29041

eFigure 1. Trends in Share of Individuals Exposed to Treatment by the Week of the Year

eFigure 2. Survival Curves on the Association Between Lifting the Eviction Moratorium and COVID-19

eFigure 3. Event Study Estimates of the Association Between Lifting the Eviction Moratorium and COVID-19, Stratified by Poverty and Rent-Burdenship Rate

eFigure 4. Event Study Estimates of the Association Between Lifting the Eviction Moratorium and COVID-19, by Sample Design

eFigure 5. Event Study Estimates of the Association Between Lifting the Eviction Moratorium and COVID-19, Stratified by Charlson Comorbidity Index and Sample Design

eFigure 6. Event Study Estimates of the Association Between Lifting the Eviction Moratorium and COVID-19, Stratified by Poverty and Rent-Burdenship Rate and Sample Design

eFigure 7. Event Study Estimates of the Association Between Lifting the Eviction Moratorium and Changing Zip Code Address

eFigure 8. Event Study Estimates of the Association Between Lifting the Eviction Moratorium and COVID-19, With and Without Z Codes

eMethods 1. Event-Time Study Model Specification

eMethods 2. Survival Curves

eReferences.

This supplemental material has been provided by the authors to give readers additional information about their work.

© 2021 Sandoval-Olascoaga S et al. *JAMA Network Open.*

eFigure 1 Trends in Share of Individuals Exposed to Treatment by the Week of the Year

Percentage

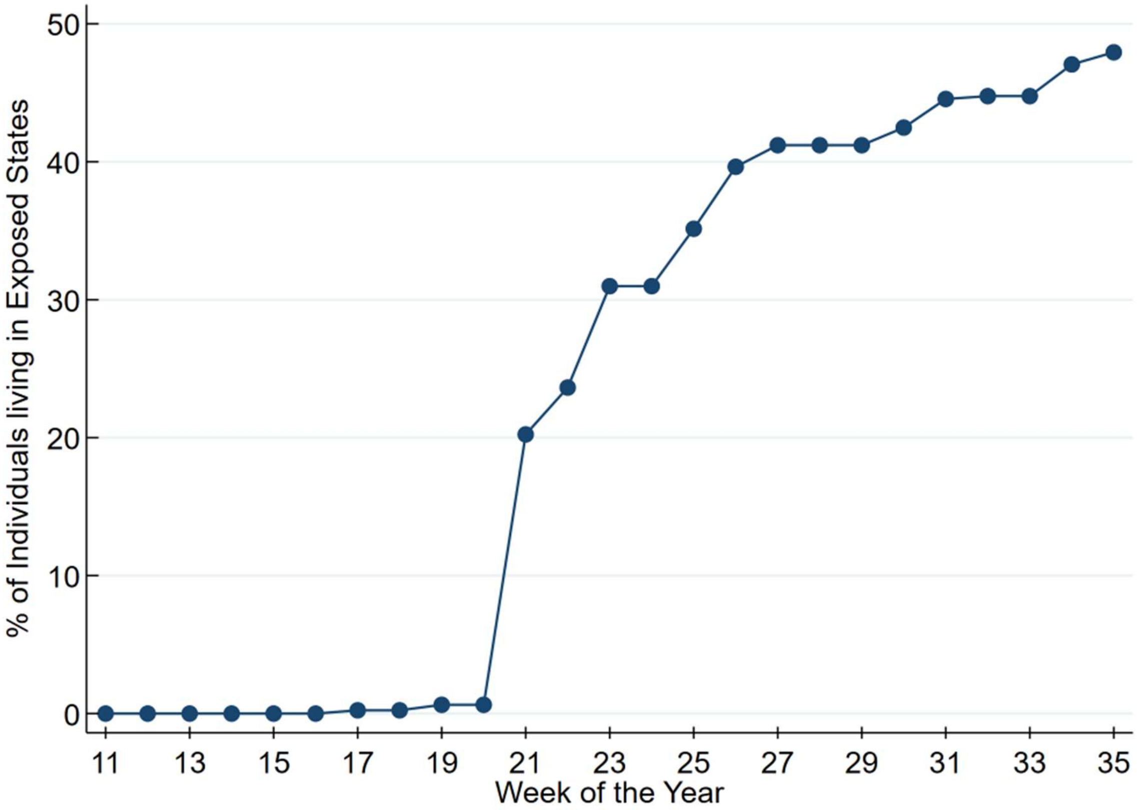

Notes: Trends in the percentage of individuals in the study sample exposed to treatment. Estimates come from the balanced sample. The y-axis shows the cumulative percentage of individuals in the study sample who lived in a state that lifted their eviction moratorium during the study period. At the beginning of the study in week 11 of 2020, no individuals were exposed to a state lifting their eviction moratorium. By the end of the study period, week 35 of 2020, 244,335 individuals lived in a state that lifted their eviction moratorium.

© 2021 Sandoval-Olascoaga S et al. *JAMA Network Open.*

eFigure 2. Survival Curves on the Association Between Lifting the Eviction Moratorium and COVID-19
Survival Probability

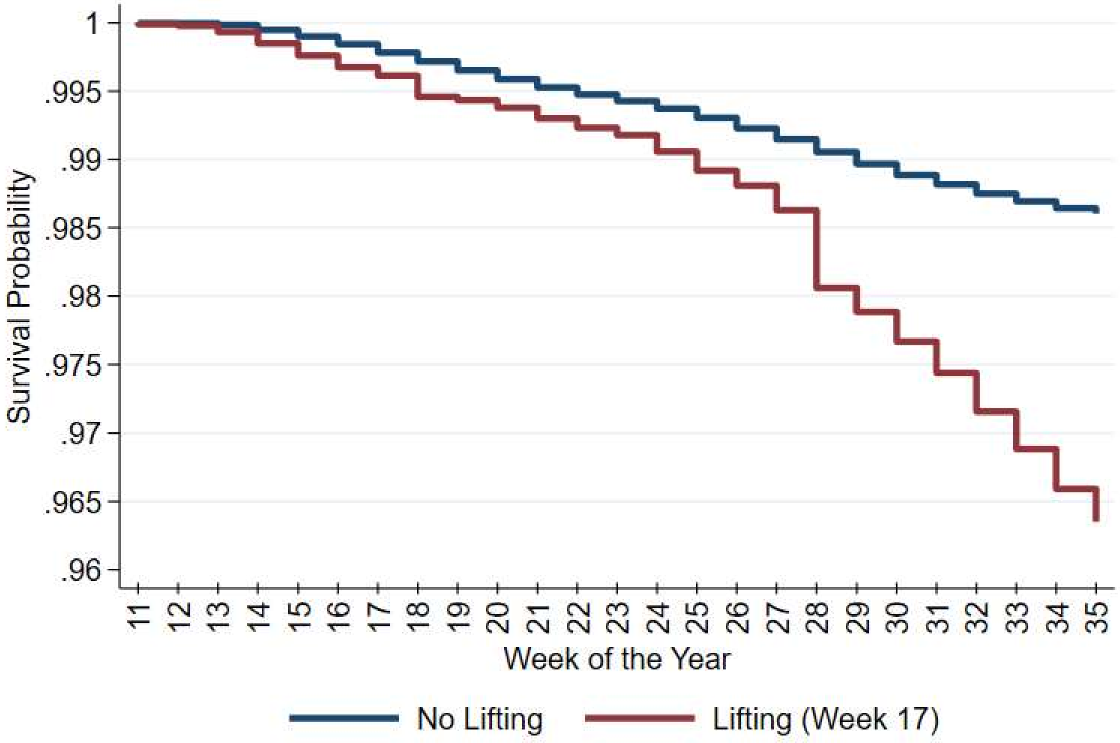

Note: Survival probability refers to the probability of remaining in the study with no diagnosis of COVID-19.

Scenario\Week	11	15	19	23	27	31	35
No Lifting	463,520	463,277	462,210	461,086	459,940	458,351	457,051
Lifting (17)	463,520	462,824	461,005	459,960	457,997	452,705	446,612

© 2021 Sandoval-Olascoaga S et al. *JAMA Network Open.*

eFigure 3. Event Study Estimates of the Association Between Lifting the Eviction Moratorium and COVID-19, Stratified by Poverty and Rent-Burdenship Rate

Hazard Ratios

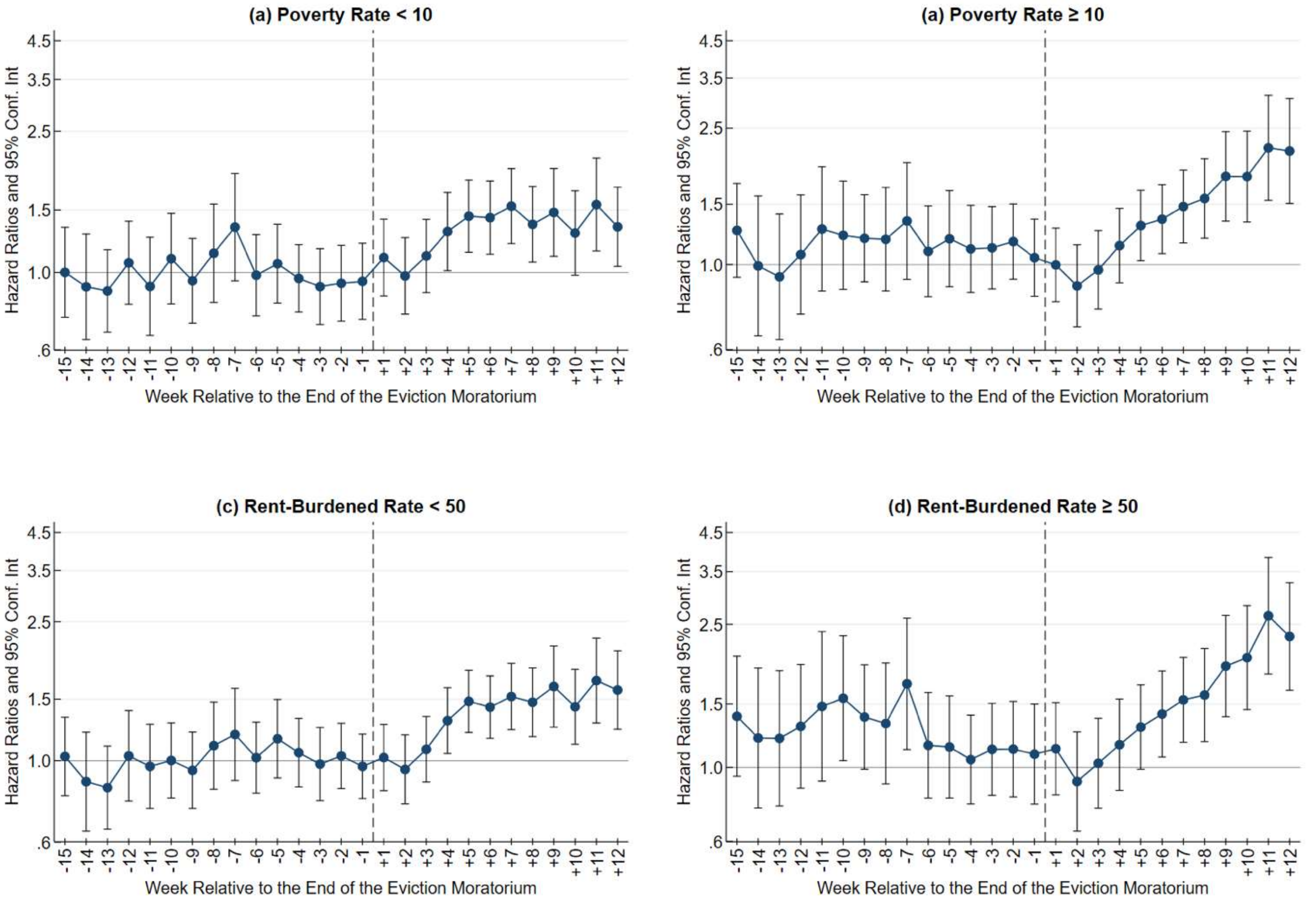

Note: Estimates come from the balanced sample. Y-axis is in log scale. The vertical dashed line represents the end of the eviction moratorium. X -axis represents the number of weeks relative to the end of the eviction moratorium. Hazard Ratios and 95% Confidence Interval from models to those estimated in Equation 1/Supplement.

© 2021 Sandoval-Olascoaga S et al. *JAMA Network Open.*

eFigure 4. Event Study Estimates of the Association Between Lifting the Eviction Moratorium and COVID-19, by Sample Design

Hazard Ratios

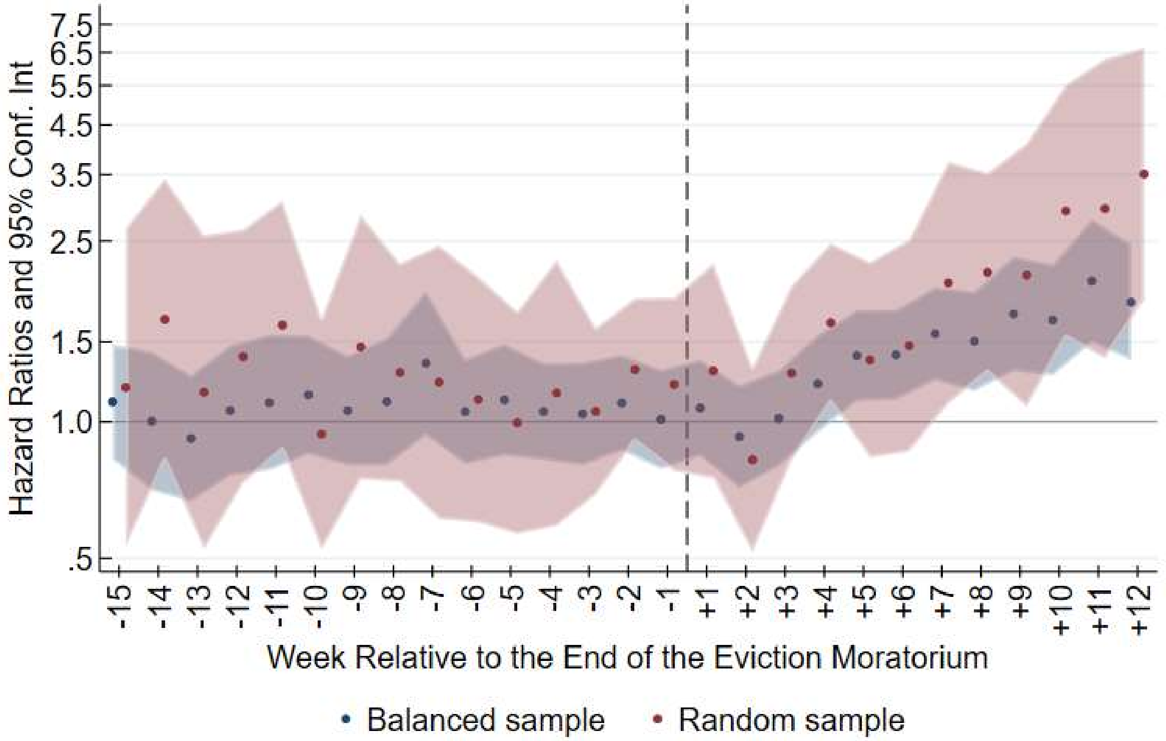

Note: Y-axis is in log scale. The vertical dashed line represents the end of the eviction moratorium. X - axis represents the number of weeks relative to the end of the eviction moratorium. Hazard Ratios and 95% Confidence Interval from models to those estimated in Equation 1/Supplement. We used, in separate models, both the balanced sample (in blue) and the 2% random sample (in red) to conduct this analysis.

© 2021 Sandoval-Olascoaga S et al. *JAMA Network Open.*

eFigure 5. Event Study Estimates of the Association Between Lifting the Eviction Moratorium and COVID-19, Stratified by Charlson Comorbidity Index and Sample Design

Hazard Ratios

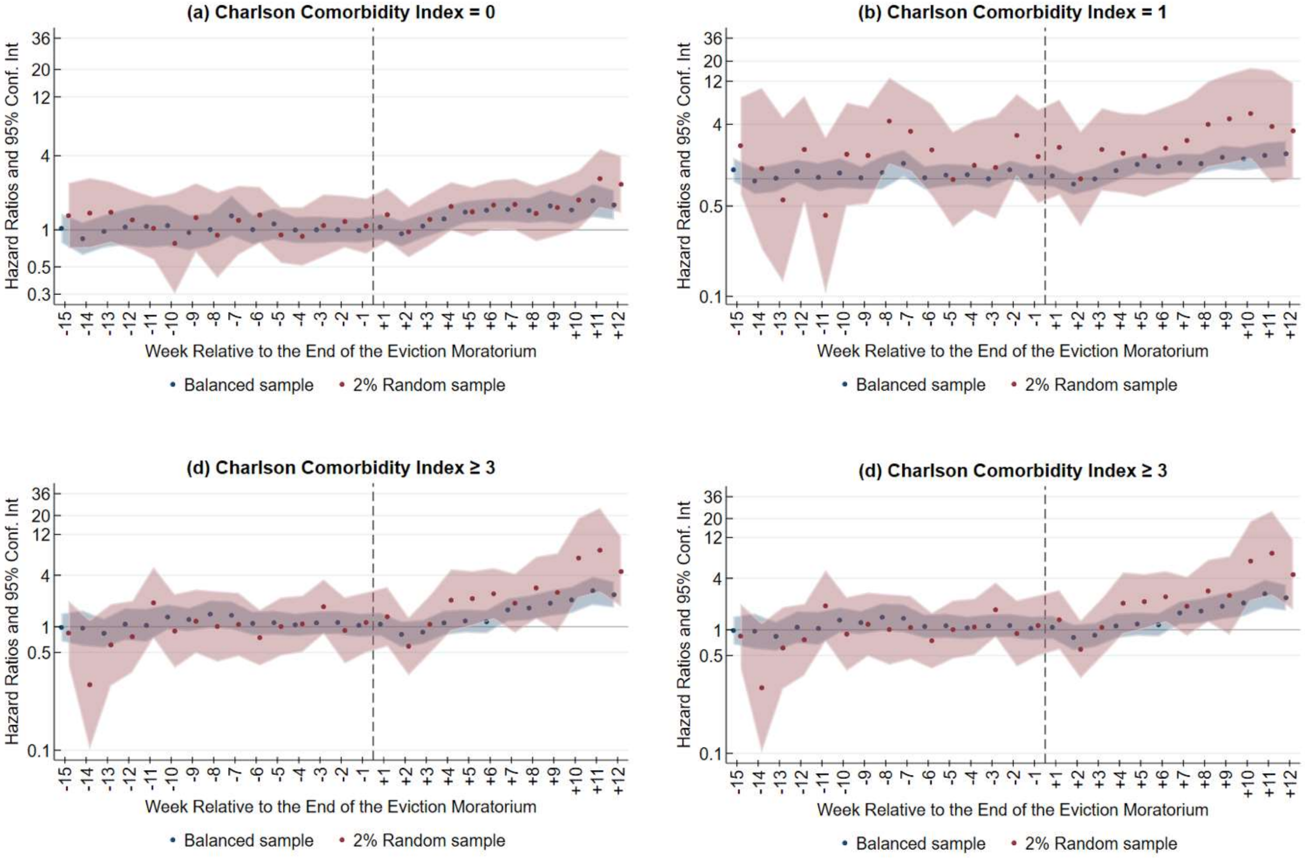

Note: Y-axis is in log scale. The vertical dashed line represents the end of the eviction moratorium. X -axis represents the number of weeks relative to the end of the eviction moratorium. Hazard Ratios and 95% Confidence Interval from models to those estimated in Equation 1/Supplement. We used, in separate models, both the balanced sample (in blue) and the 2% random sample (in red) to conduct this analysis.

© 2021 Sandoval-Olascoaga S et al. *JAMA Network Open.*

eFigure 6. Event Study Estimates of the Association Between Lifting the Eviction Moratorium and COVID-19, Stratified by Poverty and Rent-Burdenship Rate and Sample Design
Hazard Ratios

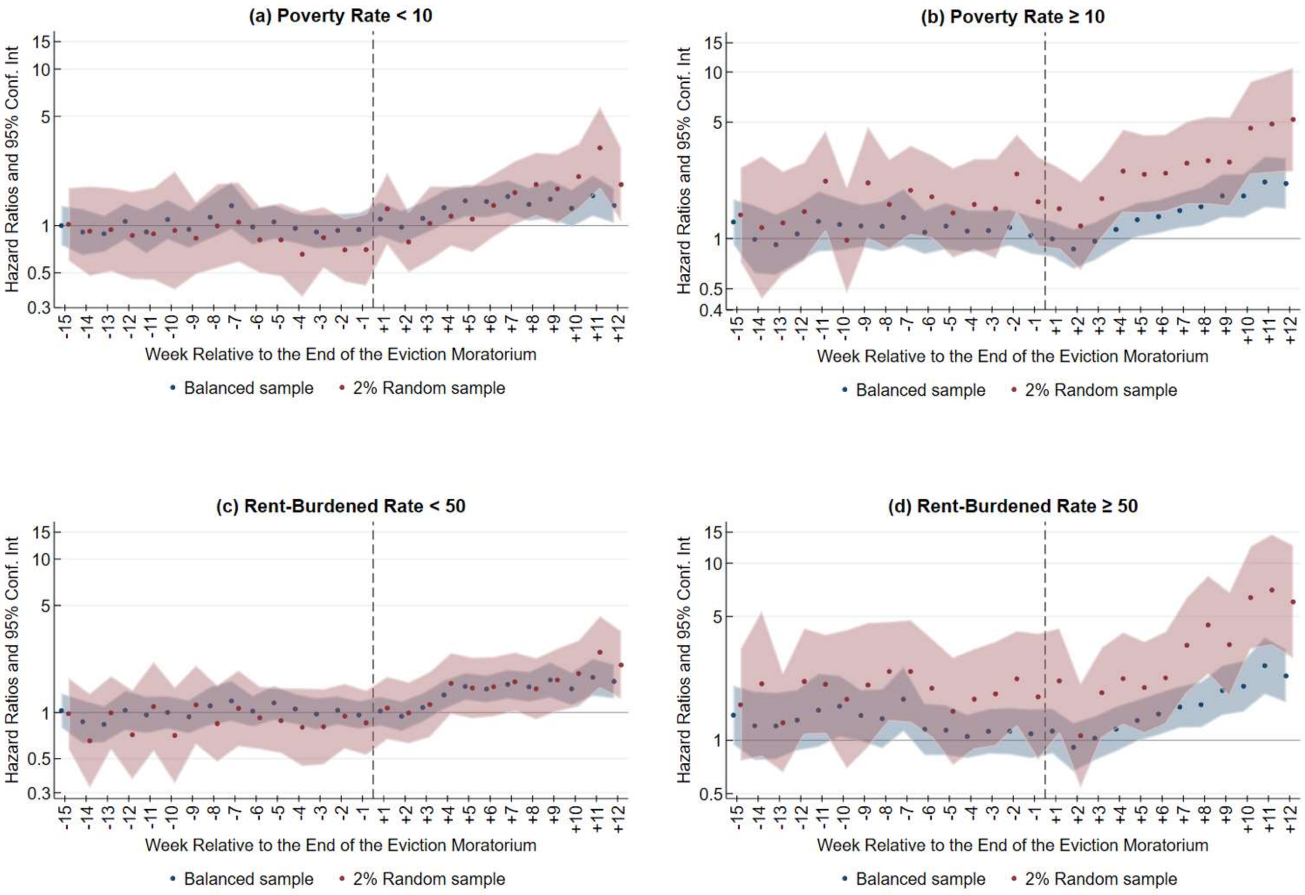

Note: Y-axis is in log scale. The vertical dashed line represents the end of the eviction moratorium. X -axis represents the number of weeks relative to the end of the eviction moratorium. Hazard Ratios and 95% Confidence Interval from models to those estimated in Equation 1/Supplement. We used, in separate models, both the balanced sample (in blue) and the 2% random sample (in red) to conduct this analysis.

eFigure 7. Event Study Estimates of the Association Between Lifting the Eviction Moratorium and Changing Zip Code Address

Hazard Ratios

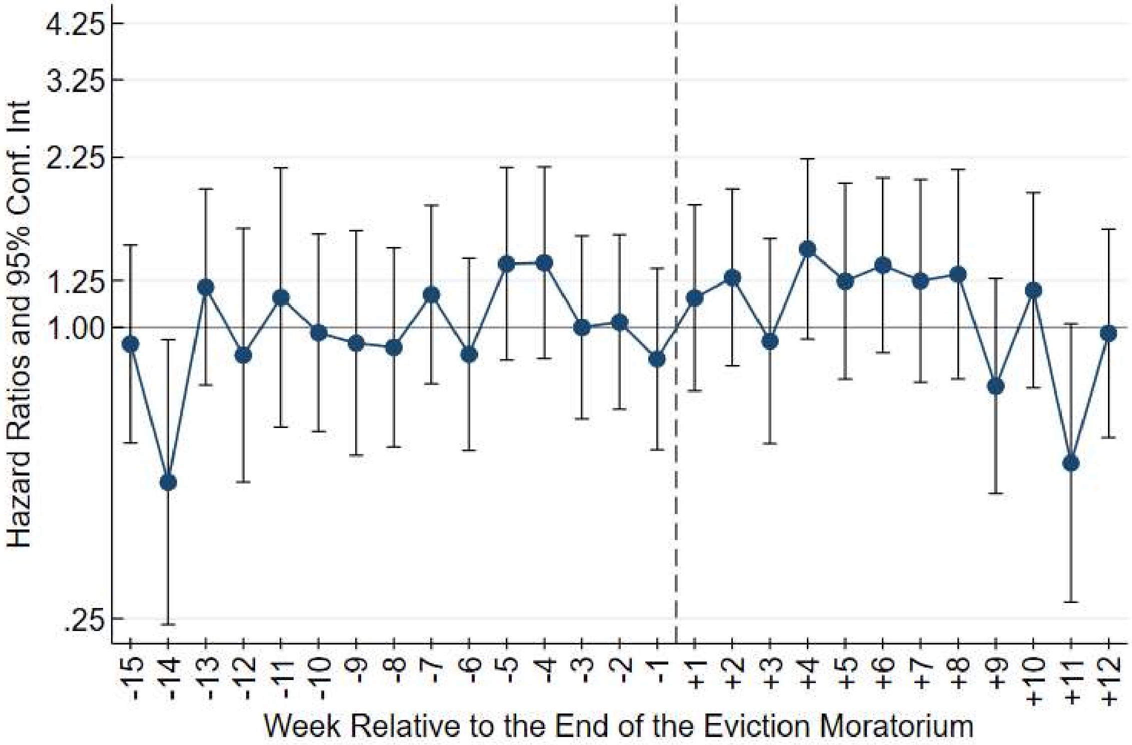

Note: Estimates come from the balanced sample. Y-axis is in log scale. The vertical dashed line represents the end of the eviction moratorium. X -axis represents the number of weeks relative to the end of the eviction moratorium. Hazard Ratios and 95% Confidence Interval from models to those estimated in Equation 1/Supplement except here the main outcome is a binary variable indicating whether the individual requested a change of residential zip code.

© 2021 Sandoval-Olascoaga S et al. *JAMA Network Open.*

eFigure 8.Event Study Estimates of the Association Between Lifting the Eviction Moratorium and COVID-19, With and Without Z Codes

Hazard Ratios

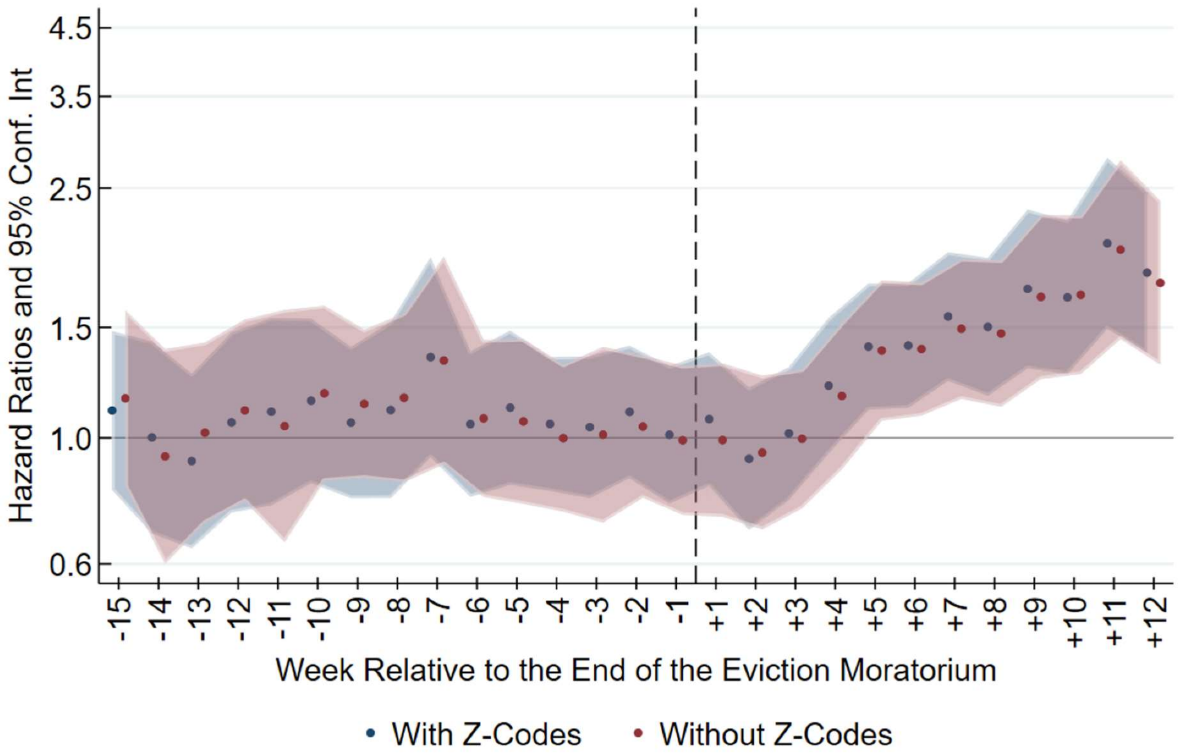

Note: Y-axis is in log scale. The vertical dashed line represents the end of the eviction moratorium. X - axis represents the number of weeks relative to the end of the eviction moratorium. Hazard Ratios and 95% Confidence Interval from models to those estimated in Equation 1/Supplement. We used, in separate models of our main specification, both with z-codes (in blue) and without z-codes (in red) to conduct this analysis.

eMethods 1. Event-Time Study Model Specification

To study the association between lifting the eviction moratorium on the hazard of being diagnosed with COVID-19 in a given week, we use a Cox regression model with time-dependent covariates in an event-time type specification.[11,12] This approach models the weekly probability of being diagnosed with COVID-19 at a given period conditional on having been observed without a positive diagnosis previously, where the treatment is defined as lifting the eviction moratorium, and treated individuals are compared to individuals living in states that had not yet lifted their moratoria.

This study uses the time from when individuals enter the study until either a COVID-19 diagnosis or time censoring at the end of the study period, just like in a classic Cox analysis. Unlike a standard Cox model, however, we also make use of information on time since the treatment occurred (i.e., lifting an eviction moratorium) for the treated. This methodology allows us to understand whether the association between expiring eviction moratoria and a COVID-19 diagnosis changes over time, which is useful when studying events that develop exponentially, such as epidemics, while also relaxing the proportional hazards assumption.

Specifically, we fitted the following model:

$$\lambda\left(t_{imt} \mid \mathbf{Z}(t) \right) = \lambda_0(t) \exp\left\{ \sum_{k=-15}^{12} \beta_k (D_{mk} \cdot T_m) + \boldsymbol{\Upsilon X}_{imt} + \boldsymbol{\Psi} M_{im} + S_m + \delta_t + \boldsymbol{u}_{imt} \right\} \quad \dots \quad (1)$$

The dependent variable $\lambda\left(t_{imt} \mid \mathbf{Z}(t) \right)$ denotes the probability that an individual, i, living in state, m, during week t is diagnosed with COVID-19. T_m is a binary variable for the treatment group, i.e., those states that implemented an eviction moratorium but lifted it. D_{mk} is a binary variable that equals 1 for those treated states during week, k, relative to the week when the state lifted their moratorium. The exposure variable is bottom coded before week 15 and top coded after week 12, implying that dynamics wear off after these points. This decision follows prior literature[11,21] to avoid difficulties when interpreting results due to sample size imbalances created by differences in the timing of lifting the moratoriums. $\boldsymbol{X}_{imt}$ is a vector of time-varying covariates (i.e., non-pharmaceutical interventions, and COVID-19 cases and tests), while M_{im} is a vector of time-invariant covariates (i.e., sex, age, type of insurance, work-industry, CCI, and z-codes diagnoses). S_m are state fixed effects that adjust for potential confounding from time-invariant state-level factors or baseline differences in socioeconomic characteristics, while δ_t are weekly fixed effects that adjust for nationwide secular trends in the outcome. u_{imt} is a vector of residuals. Standard errors are clustered at the state and week-level. We used the Breslow method for ties.

The coefficients of interest are captured by, β_k, showing the difference in outcomes for leads and lags of lifting the eviction moratoria relative to a reference week (i.e., the week a state lifted their moratorium) and relative to all states that did not lift their eviction moratorium during the reference period.

The causal identifying assumption is that COVID-19 diagnosis risk in exposed states would have continued along the same trajectories in the absence of exposure.[11] We cannot directly

© 2021 Sandoval-Olascoaga S et al. *JAMA Network Open.*

test this assumption. Still, potential violations can be probed by examining outcome trends for event weeks before lifting the eviction moratorium. We formally test this through a joint significance chi-square test simultaneously of all the terms before the eviction moratorium was lifted.

© 2021 Sandoval-Olascoaga S et al. *JAMA Network Open.*

eMethods 2. Survival Curves

To investigate temporal trends between the association of lifting an eviction moratorium and COVID-19, we fit survival curves to the data, estimating the hazard of being diagnosed with COVID-19 at time, t, for total times, T.[15] Using the time-varying outcome variable, C_t, we defined survival at t as $\Pr\,[C_t = 0]$, while the hazard at t is defined as $\Pr\,[C_t = 1 \mid C_{t-1} = 0]$. This, the survival probability at t is the product of the conditional probabilities of having survived each interval between 0 and t:[1]

$$\Pr\,[C_t = 0] = \prod_{m=1}^{t} \Pr\,[C_m = 0 \mid C_{m-1} = 0]$$

We estimated the hazards parametrically by fitting a logistic regression model for $\Pr\,[C_{t+1} = 1 \mid C_t = 0]$ that, at each, t, as:

$$\text{logit } \Pr[C_{t+1} = 1 \mid C_t = 0, D \cdot T, \boldsymbol{X}, \boldsymbol{M}, \boldsymbol{S}] =$$
$$\vartheta_{0,t} + \sum_{k=-15}^{12} \vartheta_k (D_{mk} \cdot T_m) + \vartheta_2 \sum_{k=-15}^{12} \vartheta_k (D_{mk} \cdot T_m) \cdot t + \vartheta_3 \sum_{k=-15}^{12} \vartheta_k (D_{mk} \cdot T_m) \cdot t^2 + \boldsymbol{\Upsilon X}_{imt} + \boldsymbol{\Psi} M_{im} + S_m + \boldsymbol{u}_{imt} \quad \ldots \quad (2)$$

Where, $\vartheta_{0,t}$, is a flexible time-varying function $\vartheta_{0,t} = \vartheta_0 + \vartheta_4 t + \vartheta_5 t^2$.

As in the previous analysis, T_m is a binary variable for the treatment group, i.e., those states that implemented an eviction moratorium but lifted it. D_{mk} is a binary variable that equals 1 for those treated states during week, k, relative to the week when the state lifted their moratorium. The exposure variable is bottom coded before week 15 and top coded after week 12, implying that dynamics wear off after these points. This decision follows prior literature[11,21] to avoid difficulties when interpreting results due to sample size imbalances created by differences in the timing of lifting the moratoriums. $\boldsymbol{X}_{imt}$ is a vector of time-varying covariates (i.e., non-pharmaceutical interventions, and COVID-19 cases and tests), while M_{im} is a vector of time-invariant covariates (i.e., sex, age, type of insurance, work-industry, CCI, and z-codes diagnoses). S_m are state fixed effects that adjust for potential confounding from time-invariant state-level factors or baseline differences in socioeconomic characteristics, while δ_t are weekly fixed effects that adjust for nationwide secular trends in the outcome. u_{imt} is a vector of residuals. Standard errors are clustered at the state and week-level.

We then computed estimates of the survival $\Pr\,[C_{t+1} = 0 \mid D \cdot T = d \cdot t, \boldsymbol{X} = x, \boldsymbol{M} = m, \boldsymbol{S} = s]$ by multiplying the estimates of one minus the estimates of $\Pr\,[C_{t+1} = 1 \mid C_t = 0, D \cdot T = d \cdot t, \boldsymbol{X} = x, \boldsymbol{M} = m, \boldsymbol{S} = s]$ provided by the logistic model for each individual-week. We computed two opposing counterfactual scenarios: one where every state that implemented an eviction moratorium maintained it throughout the study period, and another where every state that implemented an eviction moratorium lifted it on week 17. We chose week 17 because it was the first week a state lifted its eviction moratorium (Table 1).

We built a 95% confidence interval for the survival estimates one week before the CDC issued their eviction moratorium via bootstrapping our complete analytic sample. Given the large dataset, we conducted 50 replications for each counterfactual.

eReferences.

21. Kline P. The Impact of Juvenile Curfew Laws on Arrests of Youth and Adults. *American Law and Economics Review*. 2012;14(1):44-67. doi:10.1093/aler/ahr011

© 2021 Sandoval-Olascoaga S et al. *JAMA Network Open.*
